# Negative density-distribution relationship in butterflies

**DOI:** 10.1186/1741-7007-3-5

**Published:** 2005-03-01

**Authors:** Jussi Päivinen, Alessandro Grapputo, Veijo Kaitala, Atte Komonen, Janne S Kotiaho, Kimmo Saarinen, Niklas Wahlberg

**Affiliations:** 1 Metsähallitus, Natural Heritage Services, P.O. Box 36, 40101 Jyväskylä, Finland; 2 Department of Biological and Environmental Science, P.O. Box 35, 40014 University of Jyväskylä, Finland; 3 Department of Biological and Environmental Science, P.O. Box 65, 00014 University of Helsinki, Finland; 4 Faculty of Forest Sciences, University of Joensuu, P.O. Box 111, 80101, Joensuu, Finland; 5 Section of Natural Sciences, Jyväskylä University Museum, P.O. Box 35, 40014 University of Jyväskylä, Finland; 6 South Karelia Allergy and Environment Institute, Lääkäritie 15, 55330, Tiuruniemi, Finland; 7 Department of Zoology, Stockholm University, 106 91 Stockholm, Sweden

## Abstract

**Background:**

Because "laws of nature" do not exist in ecology, much of the foundations of community ecology rely on broad statistical generalisations. One of the strongest generalisations is the positive relationship between density and distribution within a given taxonomic assemblage; that is, locally abundant species are more widespread than locally sparse species. Several mechanisms have been proposed to create this positive relationship, and the testing of these mechanisms is attracting increasing attention.

**Results:**

We report a strong, but counterintuitive, negative relationship between density and distribution in the butterfly fauna of Finland. With an exceptionally comprehensive data set (data includes all 95 resident species in Finland and over 1.5 million individuals), we have been able to submit several of the mechanisms to powerful direct empirical testing. Without exception, we failed to find evidence for the proposed mechanisms creating a positive density-distribution relationship. On the contrary, we found that many of the mechanisms are equally able to generate a negative relationship.

**Conclusion:**

We suggest that one important determinant of density-distribution relationships is the geographical location of the study: on the edge of a distribution range, suitable habitat patches are likely to be more isolated than in the core of the range. In such a situation, only the largest and best quality patches are likely to be occupied, and these by definition can support a relatively dense population leading to a negative density-distribution relationship. Finally, we conclude that generalizations about the positive density-distribution relationship should be made more cautiously.

## Background

Species that are locally abundant tend to be more widespread than species that are locally rare [1,2]. This positive relationship between density and distribution of species has been observed in a variety of species assemblages over a spectrum of spatial scales, and it has been suggested that it may be almost an universal pattern in ecology [3-6]. However, a few studies document a negative relationship between density and distribution [7-14] (but see [15]). Only recently Gaston et al. [5] encouraged ecologists to pay more attention to the possibility of a negative relationship.

Nine mechanisms have been proposed to explain the positive relationship between density and distribution [2,3,14,16-25]. Of these, two are artefactual (sampling artefact, phylogenetic non-independence) and seven are ecological (pattern of aggregation, range position, niche breadth, resource availability, density dependent habitat selection, dispersal ability and vital rates). Negative relationship between density and distribution can be generated by similar mechanisms that give rise to a positive relationship, but for substantially different circumstances and parameter values [5,6]. Below we discuss briefly all of the mechanisms that can possibly explain the positive relationship between density and distribution, and evaluate whether they could also generate a negative relationship.

### Sampling artefact

Since low density species are less likely to be detected in surveys, a positive density-distribution relationship may result from systematic under-recording of the distribution of species that occur at low density [2,3,20]. It follows logically that sampling artefact is not expected to generate a negative density-distribution relationship [5]. However, there is a possibility that rare species with limited, but known, distribution face proportionally higher sampling effort resulting in inflated estimates of their density. When this is the case, a false negative relationship between density and distribution may be generated.

### Phylogenetic non-independence

Both positive and negative density-distribution relationship may result from related species being considered as independent data points [19,26,27]. However, phylogenetic non-independence may be the causative factor of a density-distribution relationship only if species density and distribution are determined by species specific life history characteristics affected by the common ancestry, and if there are differences between taxa in their density and distribution. Phylogenetic non-independence has been rejected as an explanation for the positive density-distribution relationship in all previous studies that have controlled its effects [5,14,25,28,29].

### Patterns of aggregation

A positive density-distribution relationship may be generated as a result of an underlying theoretical spatial distribution of individuals. For a given level of aggregation, a species with more individuals in a given area is expected to occur in more locations than a species with fewer individuals in the same area [20,24]. However, whether this purely statistical mechanism can actually cause a positive density-distribution relationship rather than serves as a restatement of the relationship in another form, is questionable [29]. This mechanism is unable to generate a negative density – distribution relationship [5].

### Range position

Empirical observations suggest that habitat occupancy and the density of individuals decline when moving along a gradient from the centre of the species geographical distribution range toward its edge [2,30-32]. Therefore, a positive density-distribution relationship in any particular region may result when species are at different positions relative to the centre of their geographical range [16]. Gaston et al. [5] argued that this mechanism cannot generate negative density-distribution relationship. More recently, Hanski [33] has pointed out that if patches are more isolated toward range edges, only the largest or best quality patches, i.e. those patches that are able to support the most dense populations, will be occupied. We note that this may lead into a negative density-distribution relationship.

### Niche breadth

Brown [2] hypothesized that a positive density-distribution relationship arises because species which have an ability to use a broader range of resources are assumed to be widespread and more abundant. However, while some evidence exists that species with greater niche breadth are more widespread [3,5,34-40], virtually all published studies fail to document a positive interspecific relationship between niche breadth and abundance (see the reviews in [5,25]). On the contrary, many species with wide niche breadth are widely distributed but locally rare [5,25]. However, even if the relationship is commonly and almost predominantly negative, it is not often significantly different from zero. Nevertheless, it seems that the relationship between niche breadth and abundance may in fact generate a negative rather than a positive density-distribution relationship.

### Resource availability

On the assumption that density and distribution of resources determine the density and distribution of the species utilizing them, a positive or a negative density-distribution relationship in the resource will generate the same relationship in the consumer [3,22]. Most of the density-distribution relationships of the resource are reported to be positive [2,41].

### Density dependent habitat selection

If a species tends to inhabit more habitats when density is high and fewer when density is low, then locally abundant species will tend to occupy more habitats and have wider distributions [17]. At present, there is little evidence for positive density-dependent habitat selection [5,25]. Instead, some evidence exists for negative density-dependent habitat selection [42-44]. A negative density-distribution relationship arises when a species has a wider distribution, i.e. inhabits more habitats, when the density is low.

### Dispersal ability

Metapopulation theory may explain both positive and negative density-distribution relationships [21]. A positive relationship may arise where a species that has high density is less likely to go extinct in a given patch than a species that has lower density, or where dispersal increases with density, thereby promoting colonization of empty patches [3,18]. Conversely, a negative relationship may emerge when the species differ in dispersal ability, since dispersal can reduce density but increase distribution [21,33].

### Vital rates

If a set of species differs only in respect to its mortality rate, then species with higher mortality will have a lower density. Moreover, the species will inhabit fewer patches than a species with low mortality, leading to a positive density-distribution [23]. Under rather restricted conditions, the vital rates mechanism could also generate a negative density-distribution relationship [23]; however, to test this mechanism, population level data on density-dependent birth and death rates are necessary [5].

Here, we will examine the relationship between density and distribution of Finnish butterflies (Hesperioidea and Papilionoidea), and test the possible mechanisms affecting this relationship. The density-distribution patterns of butterflies have been examined in many previous studies, and if a relationship was found, it was generally positive [3,14,25,38,45-47]. However, most of the studies were conducted in the British Isles, thus representing a sort of pseudoreplication in terms of range of environments and selection of butterfly species studied. Using density and distribution data from two extensive butterfly censuses, we will assess which of the four mechanisms – phylogenetic non-independence, range position, niche breadth and dispersal ability – are best at explaining the density-distribution relationship of Finnish butterflies. We will also discuss the effects of sampling artefact on the density-distribution relationship.

## Results

### Density-distribution relationships

**Figure 1:**
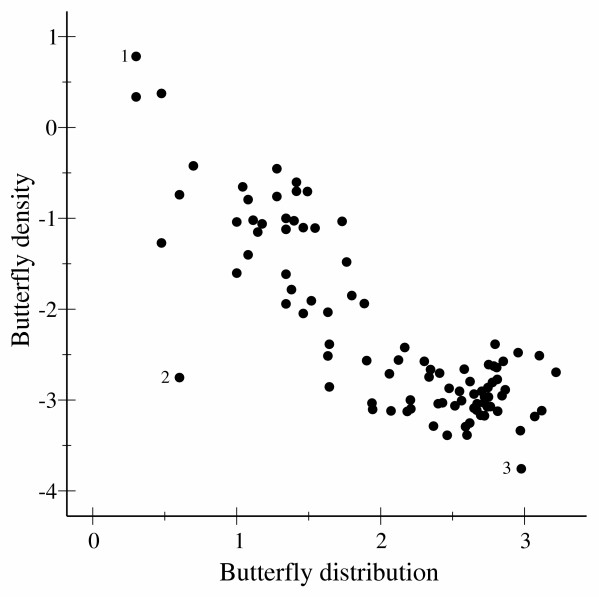
Distribution in relation to density of the Finnish butterflies. X- and Y-axes are in log10 scale. Examples of species: 1 = Agriades glandon, 2 = Argynnis laodice and 3 = Papilio machaon.

A strong negative relationship was detected between the density and distribution of Finnish butterflies (linear regression; F _1,93 _= 229.97, P < 0.001, r^2 ^= 0.71) (Fig. 1).

### Distribution of butterflies

**Figure 2:**
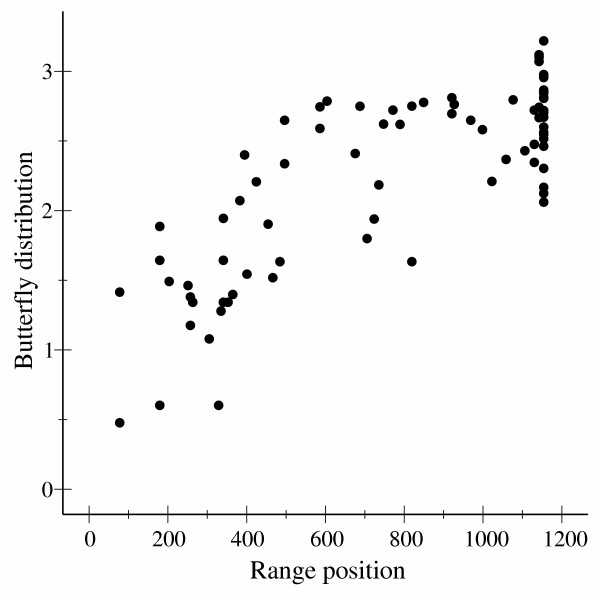
Range position in relation to distribution of the Finnish butterflies. Y-axis is in log10 scale.

**Figure 3:**
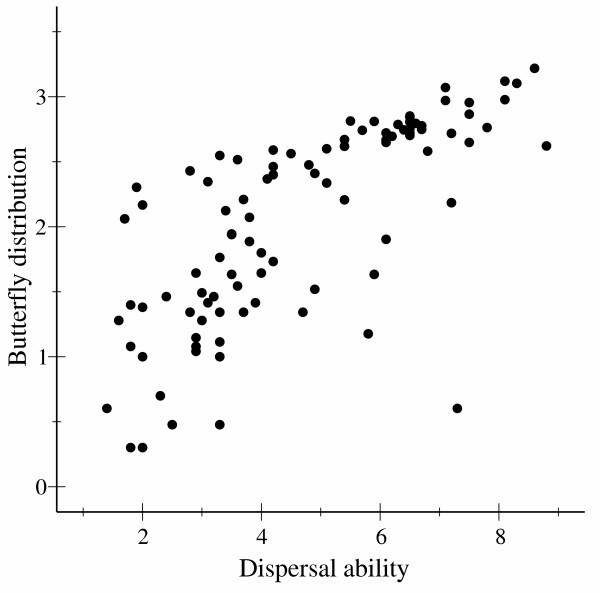
Dispersal ability in relation to distribution of the Finnish butterflies. Y-axis is in log10 scale.

To understand the effects of range position and dispersal ability on the distribution of the butterflies, we analysed the data using simple linear regression and with a multiple linear regression to even out correlated effects. In simple linear regressions, both variables had a significant positive effect on butterfly distribution (Table 1). The overall multiple linear regression was highly significant (F _2,76 _= 121.90, P < 0.001, r^2 ^= 0.76), and both of the predictor variables had an independent positive effect on the distribution of the butterflies (Table 2). The relationship between distribution of the butterflies and both of the predictor variables are shown in figures 2 and 3.

**Figure 4:**
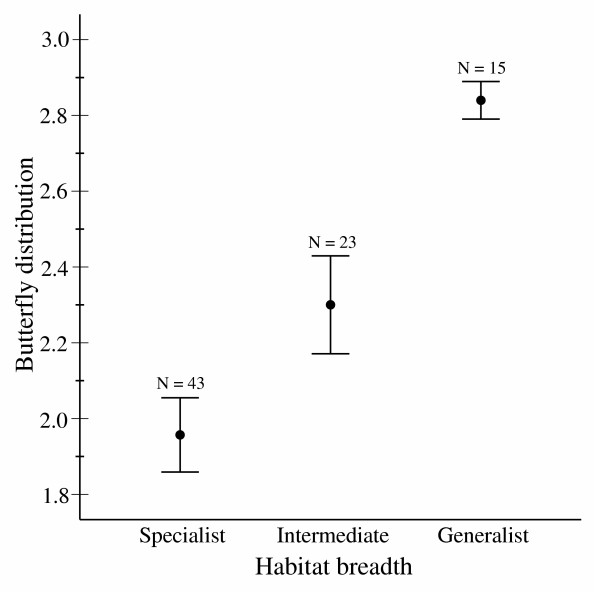
Habitat breadth in relation to distribution of the Finnish butterflies. Error bars represent mean ± 1 SE.

**Figure 5:**
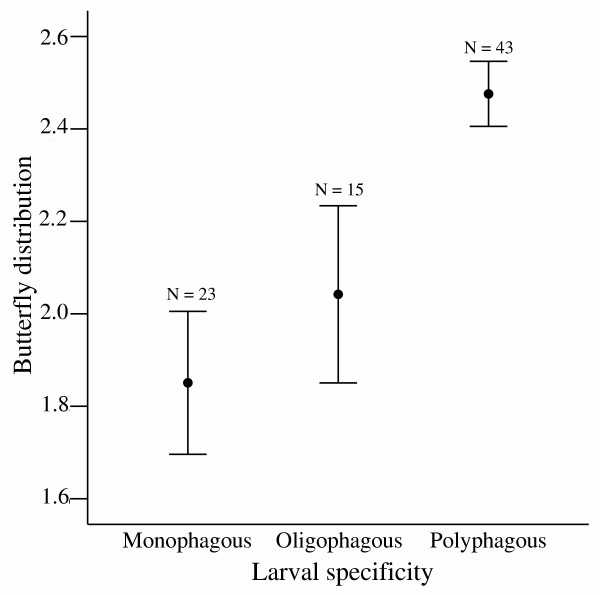
Larval specificity in relation to distribution of the Finnish butterflies. Y-axes are in log10 scale. Error bars represent mean ± 1 SE.

To study the effect of niche breadth on the distribution of the butterflies, we analysed the effects of larval specificity and adult habitat breadth on butterfly distribution using analysis of variance (ANOVA) and Tukey's multiple comparisons test. There was no significant interaction between larval specificity and habitat breadth on butterfly distribution (ANOVA F _4,72 _= 1.32, P = 0.270), and thus the interaction term was removed from the analysis. However, both larval specificity and habitat breadth had a significant effect on butterfly distribution (F _2,76 _= 8.20, P < 0.001 and F _2,76 _= 12.53, P < 0.001, respectively). A significant difference was found in the distribution of butterfly species with different habitat breadths. Specialist butterfly species had smaller distribution than generalist species (Tukey MD, mean difference = -2.03, SE = 0.40, P < 0.001), and intermediate species had smaller distribution than generalist species (Tukey MD = -1.24, SE = 0.44, P = 0.018). Specialist species tended to have smaller distributions than intermediate species, but the difference was not significant (Tukey MD = -0.79, SE = 0.35, P = 0.063; Fig. 4). No significant difference was found in the distribution of monophagous and oligophagous butterfly species (MD = -0.441, SE = 0.46, P = 0.611). However, the distribution of the polyphagous butterfly species was greater than mono- or oligo-phagous species (Tukey MD = 1.44, SE = 0.36, P < 0.001 and Tukey MD = 1.00, SE = 0.42, P = 0.051, respectively) (Fig. 5).

### Density of butterflies

**Figure 6:**
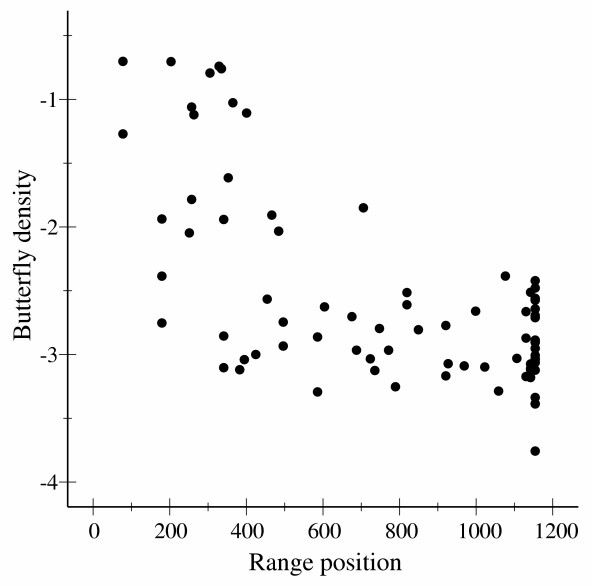
Range position in relation to density of the Finnish butterflies. Y-axis is in log10 scale.

**Figure 7:**
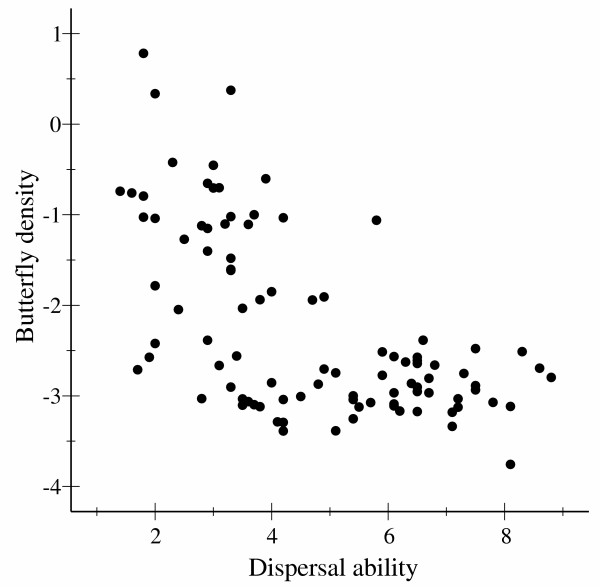
Dispersal ability in relation to density of the Finnish butterflies. Y-axis is in log10 scale.

In simple linear regressions, both range position and dispersal ability had significant negative effects on the density of the butterflies (Table 3). The overall multiple regression of the density of the butterflies on their range position and dispersal ability was highly significant (F _2,76 _= 38.84, P < 0.001; r^2 ^= 0.51), and both the predictor variables had independently negative effects on butterfly density (Table 4). The relationships between the density and the predictor variables are depicted in Figures 6 and 7.

**Figure 8:**
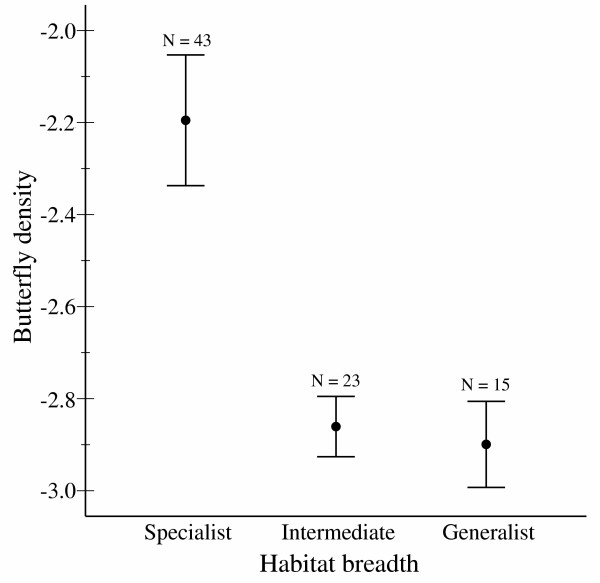
Habitat breadth in relation to density of the Finnish butterflies. Y-axes are in log10 scale.

**Figure 9:**
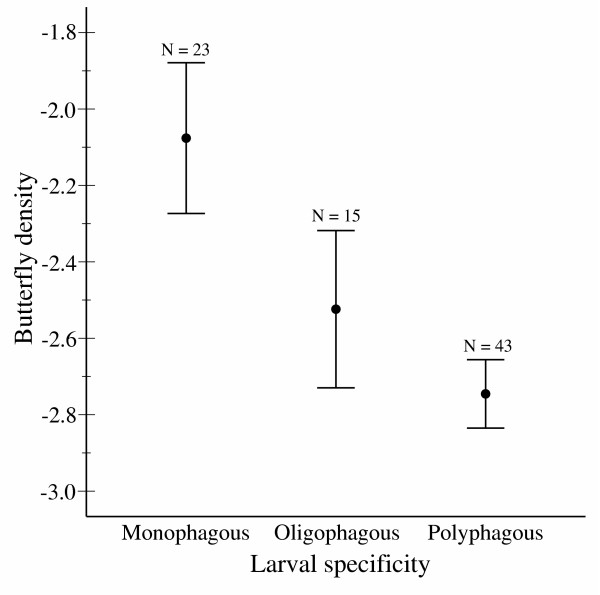
Larval specificity in relation to density of the Finnish butterflies. Y-axes are in log10 scale.

To study the effect of niche breadth on the density of the butterflies, we analysed the effects of larval specificity and adult habitat breadth by ANOVA and Tukey's multiple comparisons test. No significant interaction was observed between larval specificity and habitat breadth on butterfly density (ANOVA F _4,72 _= 0.84, P = 0.505), and thus the interaction term was removed. However, habitat breadth had a significant main effect on butterfly density (F _2,76 _= 8.49, P < 0.001). Specialist species had a greater density than intermediate (Tukey MD = 1.53, SE = 0.40, P < 0.001) or generalist species (Tukey MD = 1.62, SE = 0.49, P = 0.003), but there was no significant difference between intermediate and generalist species (Tukey MD = 0.09, SE = 0.52, P = 0.984; Fig. 8). Larval specificity also had an overall effect on butterfly density (F _2,76 _= 5.63, P = 0.005). Monophagous species had significantly greater density than polyphagous species (Tukey MD = 1.54, SE = 0.40, P < 0.001). However, Tukey test detected no differences between monophagous and oligophagous, or between oligophagous and polyphagous species (Tukey MD = 1.03, SE = 0.52, P = 0.124 and Tukey MD = 0.51, SE = 0.47, P = 0.526, respectively) (Fig. 9).

### Phylogenetic non-independence

Controlling for the phylogenetic non-independence by using the method of phylogenetically independent contrasts (CAIC) verified that none of the results reported above were artefacts of treating species as independent data points. The results with the phylogenetically independent contrasts have been tabulated in Table 5 and clearly support the previous studies on distribution, abundance or distribution-abundance relationships in which the phylogenetic non-independence not a causative factor in any of the results [5,14,25,28,29,39,40,48].

## Discussion

### Density and distribution of butterflies

A positive relationship between the density and distribution of species is expected to be an almost universal pattern in ecology [3-6]. In contrast to this, we found a strong negative relationship between density and distribution in Finnish butterflies. Here we will discuss three mechanisms-range position, niche breadth and dispersal ability – that could be responsible for generating this negative relationship.

### Range position

**Figure 10:**
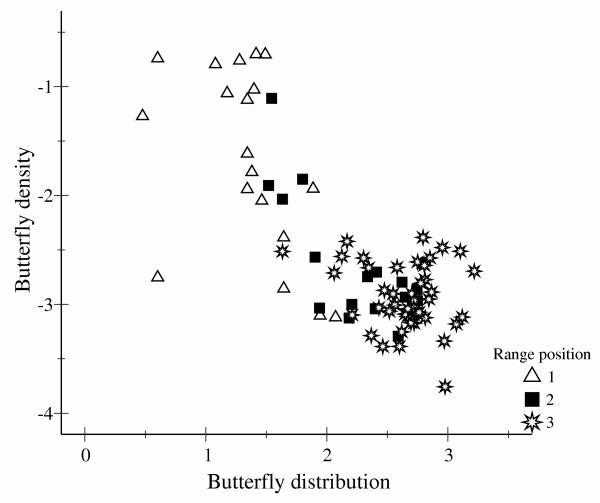
Distribution in relation to density of the Finnish butterflies. Three range position categories are separated with different symbols. The distance between the southernmost point of Finland and the northernmost observation is 1–385 km in category one, 386–770 in category two and 771–1155 in category three.

According to the range position hypothesis, species are expected to inhabit increasingly fewer localities when moving along a gradient from the centre of the species' geographical distribution range toward the edges of the range [2,31,32]. In addition, density should also decline along this gradient [2,30]. If these two assumptions are correct, a positive density-distribution relationship in any particular region will result when species are at different positions relative to the centre of their geographical ranges [16]. However, there is little evidence for the latter of the two assumptions [31,32]. Moreover, metapopulation theory suggests that if patches are more isolated toward range edges, only the largest or best quality patches will be occupied, i.e. those patches that are able to support the most dense populations [33]. If this is the case, a negative density-distribution relationship will be observed. In our study, we found a strong negative relationship between range position and density indicating that species on the edge of their geographical distribution are indeed more abundant on the patches they occupy. To illustrate the effect of this relationship on the density-distribution relationship, we divided the range position of the butterflies into three classes (1155 km / 3 = 1 – 385 km, 386 – 770 km and 771 – 1155 km) and plotted these on top of the density-distribution relationship (Fig. 10). Species on the edge of their geographical distribution (stars) had the smallest distribution and highest density, whereas the species furthest from the edge of their geographical distribution (filled boxes) had the largest distribution and lowest density. Consequently, being at different positions relative to the centre of the geographical ranges may indeed cause a negative density-distribution relationship.

### Niche breadth

**Figure 11:**
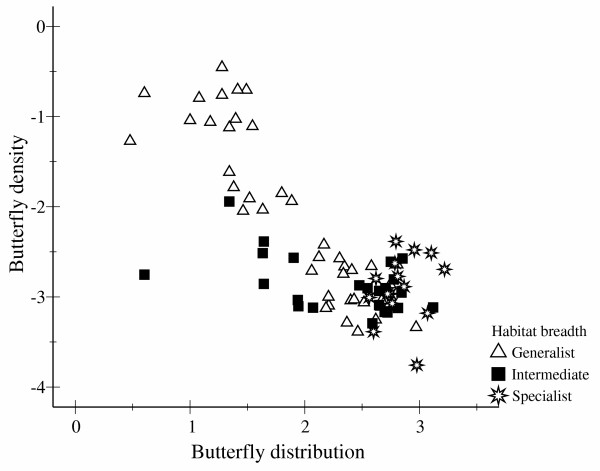
Distribution in relation to density of the Finnish butterflies. Species are classified in three categories according to their habitat breadth.

**Figure 12:**
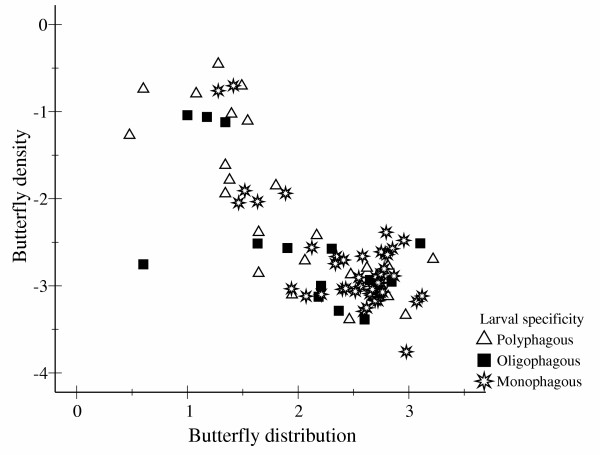
Distribution in relation to density of the Finnish butterflies. Species are divided in three categories according to their larval specificity.

The niche breadth hypothesis predicts that a positive density-distribution relationship arises because species that are able to use a broader range of resources are widespread and also have high density [2]. There is evidence of a positive relationship between niche breadth and distribution [3,5,34-40], but it is difficult to see why wider niche breadth should lead to higher density [5]. Many studies have failed to document a positive interspecific relationship between niche breadth and density (see [5,25]. In fact, most studies summarized in Gaston et al. [5] gave negative (although not statistically significant) relationships between niche breadth and density (see also [25]. In our study, we analysed niche breadth with two variables, adult habitat breadth and larval feeding specificity. We found that butterfly distribution was strongly positively related to adult habitat breadth and to larval feeding specificity, but more interestingly, both variables were significantly negatively related to density. To illustrate how these relationships may affect the density-distribution relationship, we plotted both habitat breadth (Fig. 11) and larval feeding specificity (Fig. 12) on the density-distribution relationship. These figures illustrate that the negative density-distribution relationship may be caused by differences in the niche breadth: habitat specialist species and monophagous species have higher abundance, but simultaneously have smaller distribution, than habitat generalist or polyphagous species. Based on the available empirical evidence, it seems that the relationship between distribution and niche breadth is generally positive [3,5,34-40]. If we accept this to be the case, and the density-distribution relationship is determined by niche breadth, then the form of the relationship is caused by the relationship between density and niche breadth.

### Dispersal ability

**Figure 13:**
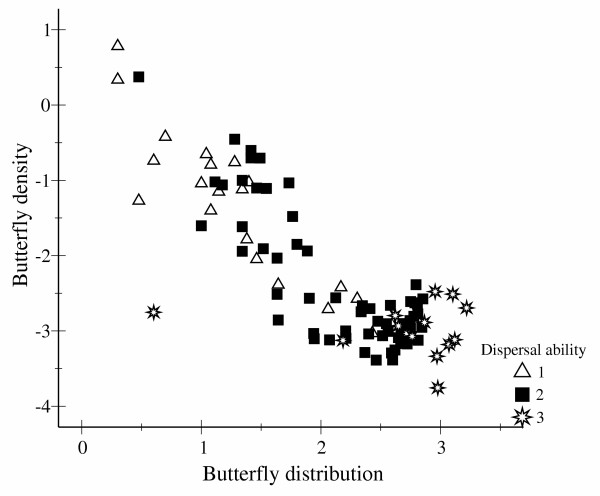
Distribution in relation to density of the Finnish butterflies. **(d) **Species are divided in three categories according the dispersal ability of the butterflies (1 = 0 – 3.33, 2 = 3.34 – 6.66 and 3 = 6.67 – 10). Note that X- and Y-axes are in log10 scale.

According to the metapopulation theory, differences in dispersal ability of species may generate negative density-distribution relationships [3,21,33]. If, for any reason, species differ in their dispersal ability and dispersal ability has a positive effect on distribution, but a negative effect on density, a negative density-distribution relationship will result. We found a strong positive relationship between dispersal ability and distribution and a strong negative relationship between dispersal ability and density, which support earlier studies in butterflies [14,25,40]. We divided the dispersal ability of the butterflies into three classes (10/3 = 0 – 3.33, 3.34 – 6.66 and 6.67 – 10) and plotted the classes on the density-distribution relationship. Fig. 13 illustrates that species with the lowest dispersal ability have the highest density but lowest distribution, while species with the highest dispersal ability have the lowest density and highest distribution, leading into the observed negative relationship between density and distribution.

## Conclusion

We conclude that many of the ecological mechanisms proposed to create a positive density-distribution relationship are also able to generate a negative relationship. Indeed, it is intriguing that many studies, which have found a positive density – distribution relationship have failed to find an ecological mechanism that would explain the observed pattern. This may not be surprising if the ecological mechanisms are more likely to generate a negative rather than positive density – distribution relationship. In support of this view we found a strong negative density-distribution relationship but what is more important also empirical support for ecological mechanisms that are able to explain the observed pattern.

Our study area (Finland) is a long, northern country extending more than 1100 km from south to north. Many of the studied butterfly species meet the edge of their distributional range in Finland. On the edge of a distribution range suitable habitat patches are likely to be more isolated than in the core of the range. In such a case, only the largest and best quality patches are likely to be occupied, but because these are the best quality patches, they may support a relatively dense population [33], leading into a negative density – distribution relationship. We suggest that one important determinant of the density-distribution relationships is the geographical position of the study area and, therefore, future studies should place greater emphasis on the comparison of the relationship in different geographical areas.

## Methods

### Data set

Our data is predominantly based on published literature [49,50] and the results of The National Butterfly Scheme in Finland (NAFI). We included all 95 butterfly species that are classified as resident or fluctuating in Finland [50] and excluded 21 species classified as migratory, irruptive or extinct [50].

### Distribution of butterflies

The distribution of butterfly species is based on the "Atlas of Finnish Macrolepidoptera" [50]. This atlas contains extensive and detailed distribution data of butterflies in Finland, covering all reliable records and observations of butterflies from 1747 to 1997 [50]. The data are compiled from many different sources including c. 1500 literature references and information extracted from several large museum and private collections [50]. The distribution data in the atlas are divided into old observations (before 1988) and new observations (1988 – 1997). We chose to describe the distribution of butterflies by the new observations, which represent the current distribution more accurately and also correspond more favourably with our density data. Moreover, there was a very strong positive correlation between distribution of butterflies based on old and new observations (Pearson correlation: r = 0.966, n = 95, P < 0.001). We describe the distribution for a given species as the number of 10 km × 10 km grid squares on the Finnish national coordinate system from which the species was recorded during the period 1988 – 1997 [50].

### Density of butterflies

The National Butterfly Scheme in Finland (NAFI) is a formal study that was established in 1991 for the purpose of monitoring the population trends of the butterflies across the country [51,52]. Monitoring studies may be liable to sampling biases [53], therefore explicit instructions were drafted in order to avoid any bias in sampling or reporting the abundance of butterflies [52]. NAFI provides quantitative abundance data including the 10 km × 10 km grid square of the Finnish national coordinate system in which the observation was made, the year, the number of individuals of each species observed and the number of observation days [52]. During the first ten-year period (1991–2000) of NAFI, 432 lepidopterologists participated in the study, providing data on 1 523 989 individuals, representing a total of 106 butterfly species.

To obtain density for each butterfly species per 10 km × 10 km grid square, we divided the total number of individuals of each butterfly species by the number of 10 km × 10 km squares occupied by the species. Some rare butterfly species with known occurrence sites may face proportionally higher sampling effort than common species [53]. To remove the effect of sampling effort on density, we divided the density by the number of observation days. Note that observation days are the days when the observer was observing at a given square, and thus include also days when a given species was not observed. The number of observation days for each species averaged 20 815, varying between 53 and 50 595 days.

It is possible that size confounds the density and distribution data if larger species are more visible and thus reported more often than smaller species. We analysed the possibility that the size of the butterfly species is a confounding factor. There is practically no dimorphism between the sizes of male and female butterflies. Therefore, as a size measure we used only the female wing span reported in Marttila et al. [49], in which the mean of a sample of 20 females was reported with an exception of some rare species with fewer individuals measured. We found no evidence of any size bias: there was no relationship between the female wing span and the density or distribution of the species (linear regression F _1,93 _= 0.04; P = 0.835, r^2 ^= 0.00 and F _1,93 _= 0.99; P = 0.987, r^2 ^= 0.00, respectively).

### Range position

To determine the range position, we measured the distance (km) between the northernmost distribution record and the southernmost point (Hankoniemi) of Finland from the maps in Huldén et al. (2000). Note that the longest possible range was 1155 km. In analysis including range position, we only included species, the distribution range of which begins from southern Finland [50]. Thus, we excluded the species which occur only in Northern Finland (n = 14, [49]), and two species (*Lycaena helle *and *Clossiana thore*) which do not range to southern Finland.

### Niche breadth

We describe the niche breadth for each butterfly species using two different measures: 1) larval host plant specificity, and 2) the number of habitat types occupied by the adults. We excluded from this analysis the species which occur only in Northern Finland (n = 14, [49]). This was done because their larval host plants are unknown or are not confirmed and because the habitat types of Northern Finland are unique compared to other Finnish habitat types.

The data on larval host plant specificity in Finland are based on Huldén et al. [50] and Wahlberg [54]. We classified the larval host plant specificity into three classes: (1) monophages, i.e. species that feed on a single plant species; (2) oligophages, i.e. species that are restricted to one genus of food plants; and (3) polyphages, i.e., species that feed on one or more than one family of food plants.

The main habitats that Finnish butterflies occupy have been categorised into four types [49]: (1) uncultivable lands (e.g. edge zones beside industrial area, harbour and storage areas, loading places, uncropped fields, and other unbuilt areas which have been exposed to human impact), (2) meadows (many kinds of non-cultivated open grasslands), (3) forest edges (e.g. road sides), and (4) bogs. Adult niche breadth is the number of habitat types in which the adults typically are found. As there were only two species (*Pieris napi *and *Gonepteryx rhamni*) that occupied all four main habitat types, habitat types three and four were combined. Value one represents specialist species that are limited to one habitat type, value two represents intermediate species that occur in two habitat types, and value three represents generalist species that occur in three or four habitat types.

### Dispersal ability

Metapopulation theory predicts that species with greater dispersal ability are likely to occupy more habitat patches than species with lower dispersal ability [3]. To describe the relative dispersal ability of butterfly species, we adopted the method described in Cowley et al. [14]. We sent a questionnaire to experienced lepidopterists in Finland and asked them to give a "dispersal ability index" (0–10) for each butterfly species. In the questionnaire, a zero value indicates that a given butterfly species is extremely sedentary and a value of ten means that it is extremely mobile. To obtain a relative dispersal ability for each butterfly species, we calculated the average dispersal ability from returned questionnaires (n = 13).

To ensure our measure of dispersal ability is reliable, we compared our measure with the dispersal ability estimated previously by Bink [55] (nine dispersal ability classes), Pollard and Yates [56] (three dispersal ability classes based on mark-release-recapture studies), Cowley et al. [14] (continuous variable based on questionnaires) and Cook, Dennis & Hardy [57] (continuous variable based on vagrancy in grids). The correlations between our measure and those of Bink [55], Pollard and Yates [56], Cowley et al. [14] and Cook et al. [57] were all strongly positive and significant (Pearson correlation; r = 0.672, n = 73, P < 0.001, ANOVA; F_3,27 _= 8.74, r = 0.567, P < 0.001, Pearson correlation; r = 0.703, n = 31, P < 0.001 and Pearson correlation r = 0.602, n = 11, P = 0.050, respectively), indicating that our dispersal ability is in line with other independent estimates and thus reliable (see also [58]).

### Phylogenetic non-independence

**Figure 14:**
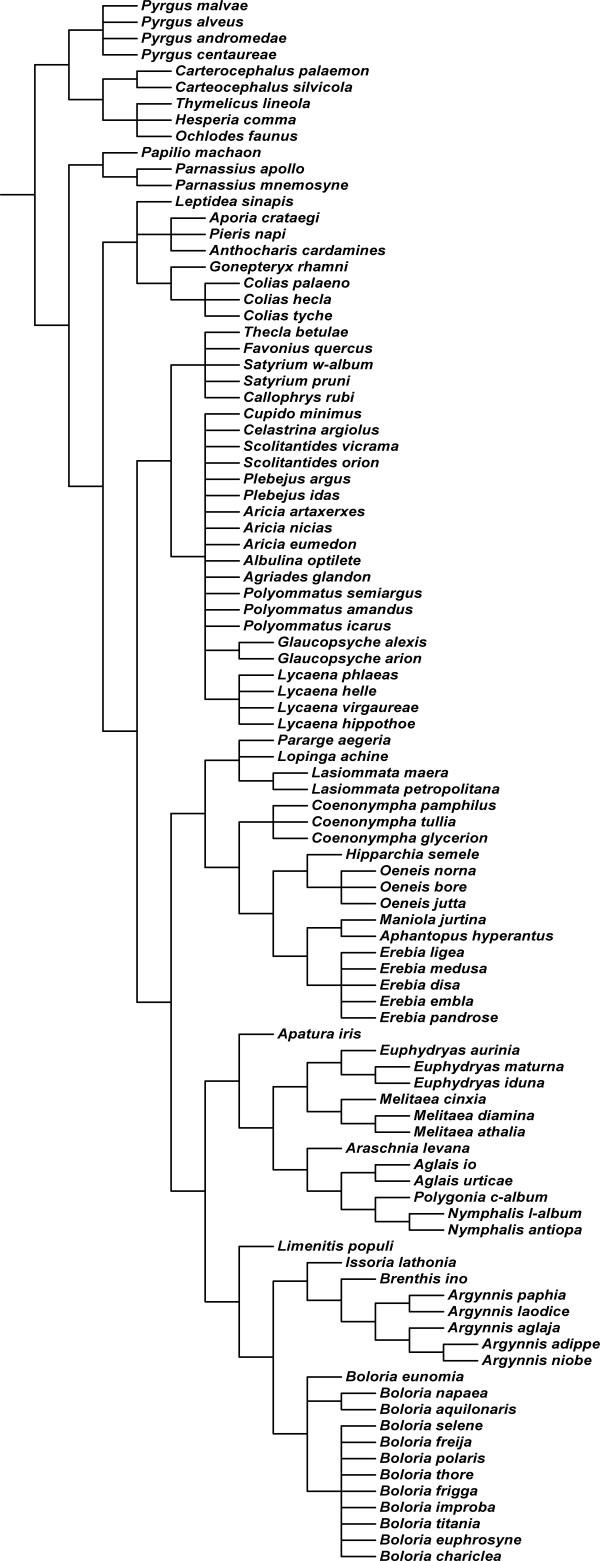
The phylogeny of butterflies used in this study. The relationships of groups are based on a number of published studies (see text for details).

Lack of statistical independence among species for the traits of interest was tested using the method of phylogenetically independent contrasts [19] as implemented in the CAIC program [59]. Statistical control of phylogenetic non-independence requires knowledge of the phylogeny [19,27]. However, knowledge of the general phylogenetic relationships among butterfly species is still in a state of flux [60], and there are no studies available that look explicitly at the relationships of species in Finland. However, the recent surge of published studies on various groups of butterflies allows us to compile a likely phylogeny for Finnish butterflies (Fig. 14). We took the relationships of the butterfly families from [60], the relationships within Papilionidae from [61], and the relationships within Nymphalidae from various sources [62-65]. Relationships within Pieridae, Lycaenidae and Hesperiidae are based on current taxonomy, with morphologically well-defined groups shown as monophyletic. In the analysis all branch length were assumed equal because no estimate of evolutionary distance exist for the entire data set. However this option is justified under the assumption of punctuated evolution. Regression analysis was used to investigate the standardized linear contrasts calculated by CAIC [19]. Note that the regression lines must pass though the origin [66,67].

## Authors' contributions

JP, VK, AK and JSK compiled and analysed most of the data, and wrote the manuscript. NW constructed the phylogeny and AG conducted the phylogenetic analyses. KS provided the density data.

## Figures and Tables

**Table 1:** Simple linear regressions for the distribution of butterflies.

	Slope	SE	t	df	P
Range position	0.00	0.00	11.29	77	< 0.001
Dispersal ability	0.65	0.06	10.15	93	< 0.001

**Table 2:** Multiple linear regression for the distribution of butterflies.

	Slope	SE	t	df	P
Range position	0.00	0.00	10.04	76	< 0.001
Dispersal ability	0.33	0.05	6.66	76	< 0.001

**Table 3:** Simple linear regressions for the density of butterflies.

	Slope	SE	t	df	P
Range position	-0.00	0.00	-7.51	77	< 0.001
Dispersal ability	-0.73	0.10	-7.47	93	< 0.001

**Table 4:** Multiple linear regression for the density of butterflies.

	Slope	SE	t	df	P
Range position	-0.00	0.00	-5.83	76	<0.001
Dispersal ability	-0.28	0.08	-3.56	76	<0.001

**Table 5:** Linear regression results between all variables after controlling for the phylogenetic non-independence. N is the number of independent contrasts.

*Dependent variable*	*Independent variable*	*N*	*Slope*	*r*^2^	*P*
Density	Distribution	45	-1.13	0.67	0.000
Distribution	Body size	45	-0.72	0.01	0.513
Distribution	Range position	42	1.62	0.58	0.000
Distribution	Dispersal ability	45	0.80	0.76	0.000
Distribution	Larval specificity	14	0.46	0.55	0.002
Distribution	Habitat breadth	17	0.47	0.48	0.002
Density	Body size	45	1.92	0.03	0.232
Density	Range position	42	-1.90	0.30	0.000
Density	Dispersal ability	45	-1.04	0.54	0.000
Density	Larval specificity	14	-0.51	0.59	0.000
Density	Habitat breadth	17	-0.71	0.45	0.002
